# Awareness and Attitudes Regarding Adverse Drug Events and Reporting in South Africa

**DOI:** 10.1007/s43441-025-00795-x

**Published:** 2025-05-13

**Authors:** Nokukhanya Ncube, Martha S. Lubbe, Hanlie Steyn, Nkengafac V. Motaze

**Affiliations:** Medicine Usage in South Africa, Faculty of Health Sciences, North-West University, Potchefstroom, South Africa

**Keywords:** Adverse drug events, Drug side-effects, Reporting, Consumer, Pharmacovigilance

## Abstract

**Background:**

Reporting of adverse drug events (ADEs), by consumers enhances medication-related risk surveillance, public awareness, and understanding of medicine safety. The aim of this study was to explore adults’ awareness of ADEs, attitudes towards reporting and perceptions of their role in reporting ADEs in South Africa.

**Method:**

We conducted a cross-sectional, analytical study in which adults residing in South Africa completed an online questionnaire. The data collected was analysed using both descriptive and inferential statistics.

**Results:**

We received responses from 350 participants. Most participants (86.2%, *n* = 302; *N* = 350) reported having heard about ADEs and the majority of participants (94.4%, *n* = 301; *N* = 319) indicated that reporting of ADEs was important. The Med Safety App was not widely known (17.3%, *n* = 58; *N* = 336) while the South African Health Products Regulatory Authority (SAHPRA) was relatively well known (77.4%, *n* = 260; *N* = 336). Healthcare providers only educated 55.7% (*n* = 180; *N* = 323) of the participants about ADEs and only 50.5% (*n* = 163; *N* = 323) of the participants asked their healthcare providers about ADEs. Awareness regarding ADEs was significantly higher (*p* < 0.001) among healthcare professionals (HCPs) compared to non-healthcare professionals (non-HCP).

**Conclusion:**

Most participants were aware of ADEs and agreed it was important to report ADEs although reporting tools, such as the Med Safety App, were not well known. We recommend awareness campaigns on reporting processes because this could improve consumer reporting of ADEs in South Africa.

## Introduction

Medicines are of great importance because they are used to treat and prevent a range of diseases. However, the use of medicines is not without challenges. It is important to monitor the adverse effects that are experienced in order to better understand the safety and risk-benefit of medicinal products. Therefore, systems should be in place to monitor and report any suspected adverse drug events (ADEs) [1].

Spontaneous reporting of ADEs to pharmacovigilance centres is the main source of post-marketing data on medicine safety [2]. These reports can be received from various sources, including medicine users or consumers, healthcare professionals (HCPs), the pharmaceutical industry and medical literature [3].

In recent years, there has been an increase in direct consumer reporting of ADEs to national pharmacovigilance centres and this includes reports submitted directly by patients or their relatives [4]. Consumer reporting offers additional perspectives on people’s experiences with ADEs that are unavailable from the HCP reports. With increased access to the internet and social media, consumers can play a significant role in pharmacovigilance; by reporting ADEs, they provide data on safety of medicines in addition to the data provided by HCPs [5].

To allow for consumer involvement in pharmacovigilance, they must be aware of what needs to be reported, who to report to, and have easy access to the reporting tools [6]. Therefore, awareness campaigns aimed at increasing consumer reporting, which focuses on both consumers and HCPs, must be undertaken [7].

South Africa became the first African nation to join the World Health Organization’s Programme for International Drug Monitoring as a full member in 1992 [8]. As a member state of this programme, South Africa must conduct pharmacovigilance activities in the country including the establishment of a system to collect, document, and evaluate ADE reports [9].The Medicines and Related Substances Act, 1965 (Act No. 101 of 1965) [10], as amended, requires the South African Health Products Regulatory Authority (SAHPRA) to oversee the use of all health products in South Africa. The vigilance unit of SAHPRA in cooperation with the National Department of Health (NDoH), serves as the central coordinator of pharmacovigilance activities in South Africa [11]. Despite the existence of these systems, under-reporting of ADEs remains a challenge in South Africa [12].

Under-reporting of ADEs is a challenge worldwide [13] which has not been resolved by the existence of reporting tools such as FAERS in the United States of America [14], the Yellow Card Scheme in the United Kingdom [15] and VigiBase, the World Health Organization (WHO) database of spontaneous safety reports [16].

The Med Safety App is a mobile application created to simplify and make more effective, the reporting of suspected ADEs, including adverse events after immunization (AEFIs). The application was introduced by SAHPRA in 2021 as part of its initiatives to improve reporting of side effects of health products. This application allows both the public and HCPs to learn about medicine safety from SAHPRA, and raises awareness about medicines, their possible side effects, and pharmacovigilance [17].

The aim of this study was to evaluate adults’ knowledge of pharmacovigilance, attitudes toward reporting ADEs, and perceptions of their role in reporting ADEs in South Africa. The knowledge gained from this study will provide information that may help guide the actions needed to promote ADE reporting and ultimately improve medicine safety in South Africa.

## Materials and Methods

An analytical, cross-sectional study was conducted in which a self-administered, online, structured questionnaire was created using questionnaires from published studies with comparable objectives [18–22]. A statistician evaluated the questionnaire’s face validity, and experts from the Medicine Usage in South Africa Scientific Committee evaluated its relationship to the study’s objectives. The same questionnaire was completed by each participant. The questionnaire contained 32 questions organised into: Section A: Socio-demographic information; Section B: Awareness of adverse drug events; Section C: Attitudes regarding adverse drug events and adverse drug events reporting; Section D: Practices and experience with reporting adverse drug events; Section E: Barriers to reporting adverse drug events. Sections A, B and C of the questionnaire are presented in this paper.

To recruit participants, a news article [23] was published on the English News24 and Afrikaans Netwerk24 online platforms that contained a link to SurveyMonkey^®^ [24] to complete the survey. The News24 and Netwerk24 domains collectively attract on average 18,573,335 daily active users [25]. The survey was available for data collection from 18 April 2023 to 18 June 2023. To participate in this study participants had to be at least 18 years old, live in South Africa, and give their consent. Due to the recruitment strategy employed in this study, the response rate could not be determined.

The statistical analysis for both descriptive (numbers and or percentages) and inferential statistics was carried out using R statistical software version 4.3.1 [26]. In addition, Pearson’s chi-square test or Fischer’s test was used, when appropriate, to test for an association between two categorical variables. The Wilcoxon rank sum test was used to compare median ages between groups of participants. All statistical tests were two-tailed, and the type-I error rate was set at 5% (α = 0.05).

## Results

A total of 374 responses were retrieved from SurveyMonkey^®^ and 350 (93.6%) records were analysed after exclusion of records with missing age, those who only completed the demographic section and participants residing outside South Africa.

### Participants’ Characteristics

**Fig. 1:**
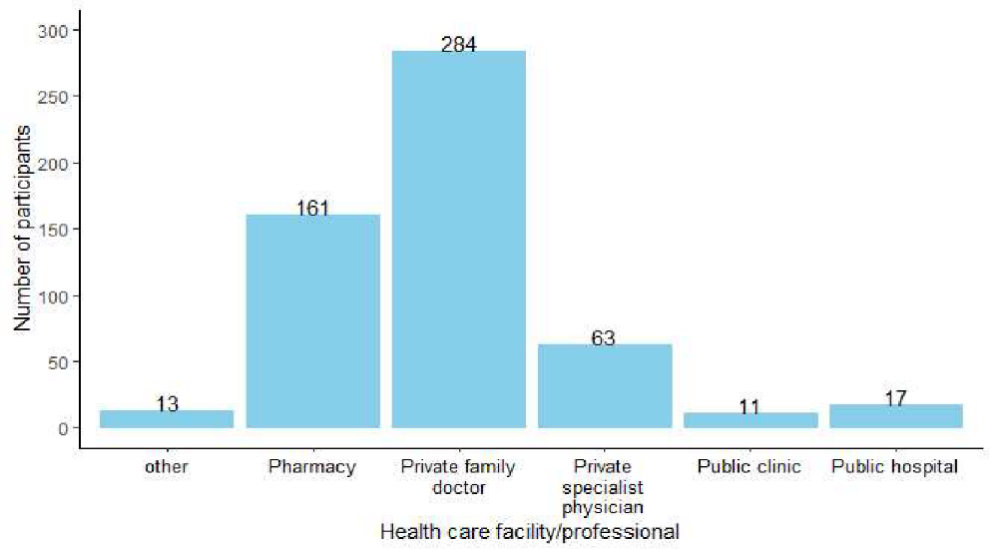
HCPs consulted for health-related needs

The median age (IQR) of participants was 52 (38, 62) and the majority of participants (71.4%, *n* = 250; *N* = 350) were female. The majority of participants, 86.0% (*n* = 301; *N* = 350) had tertiary education and only 0.3% (*n* = 1; *N* = 350) reported having no formal education. All provinces of South Africa were represented in the study, with the majority of the participants residing in Gauteng (42.6%, *n* = 149; *N* = 350), Western Cape (22.0%, *n* = 77; *N* = 350), North West (9.4%, *n* = 33; *N* = 350) and KwaZulu-Natal (9.1%, *n* = 32; *N* = 350). Most participants (91.4%, *n* = 320; *N* = 350) lived in urban areas. Participant characteristics are provided in Table 1. The majority of the participants indicated that they visit their private family doctors to seek treatment for general health problems (Fig. 1).

**Table 1:** Participant characteristics

Characteristic	Overall, *N* = 350^*1*^	Non-HCP *N* = 248^*1*^	HCP *N* = 102^*1*^	*p*-value^2^
**Median Age** ^1^	52 (38–62)	53 (41–64)	46 (34–59)	0.002
**Age Range (years)**				0.014
19–30	40 (11.4%)	26 (10.5%)	14 (13.7%)	
31–40	61 (17.4%)	34 (13.7%)	27 (26.5%)	
41–50	66 (18.9%)	45 (18.1%)	21 (20.6%)	
51–60	86 (24.6%)	67 (27.0%)	19 (18.6%)	
61 and above	97 (27.7%)	76 (30.6%)	21 (20.6%)	
**Sex**				0.004
Female	250 (71.4%)	166 (66.9%)	84 (82.4%)	
Male	100 (28.6%)	82 (33.1%)	18 (17.6%)	
**Province**				-
Eastern Cape	13 (3.7%)	7 (2.8%)	6 (5.9%)	
Free State	17 (4.9%)	13 (5.2%)	4 (3.9%)	
Gauteng	149 (42.6%)	110 (44.4%)	39 (38.2%)	
KwaZulu-Natal	32 (9.1%)	21 (8.5%)	11 (10.8%)	
Limpopo	8 (2.3%)	3 (1.2%)	5 (4.9%)	
Mpumalanga	17 (4.9%)	12 (4.8%)	5 (4.9%)	
North West	33 (9.4%)	19 (7.7%)	14 (13.7%)	
Northern Cape	4 (1.1%)	4 (1.6%)	0 (0.0%)	
Western Cape	77 (22.0%)	59 (23.8%)	18 (17.6%)	
**Level of** **education**				< 0.001
No formal education	1 (0.3%)	1 (0.4%)	0 (0.0%)	
Primary school	1 (0.3%)	1 (0.4%)	0 (0.0%)	
Secondary school	47 (13.4%)	44(17.7%)	3 (2.9%)	
Tertiary education	301 (86.0%)	202 (81.5%)	99 (97.1%)	
**Area of** **residence**				0.072
Rural	30 (8.6%)	17 (6.9%)	13 (12.7%)	
Urban	320 (91.4%)	231 (93.1%)	89 (87.3%)	

^*1*^ Median (IQR); n (%)

^*2*^ Wilcoxon rank sum test; Pearson’s Chi-squared test; Fisher’s exact test

*Note* HCP denotes participants who reported that they were healthcare professionals or who were enrolled in programs to become HCPs. Non- HCP refers to participants who are not healthcare professionals

**Fig. 2:**
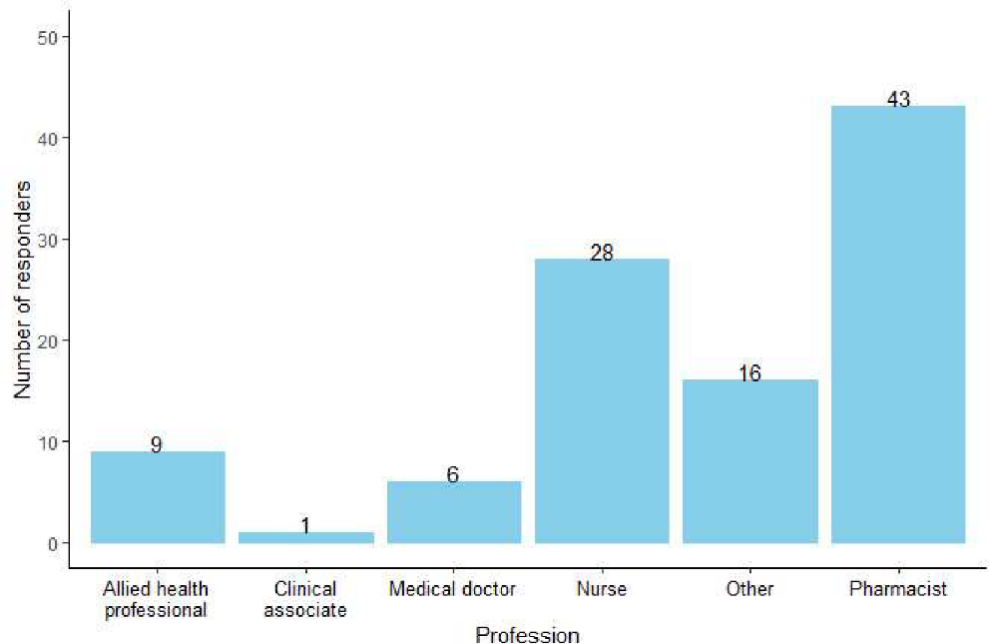
Professions of healthcare workers. The category referred to as “other” includes fields such as epidemiologist, medical scientist and paramedics

Out of 350 participants, 29.1% (*n* = 102; *N* = 350) reported that they were HCPs or currently studying to be HCPs, with pharmacists representing the majority of these participants (42.2%, *n* = 43; *N* = 102). The distribution by field of practice is shown in Fig. 2. To account for participants’ medical training, the responses from the HCPs and non-HCPs will be reported separately where applicable.

### Awareness of ADE

Of the 350 participants 302 (86.3%) had heard of ADEs, including 98.0% (*n* = 100; *N* = 102) of HCPs and 81.5% (*n* = 202; *N* = 248) of non-HCPs (*p* < 0.001). Among those who had heard of ADEs, 94.8% (*n* = 276; *N* = 291) of participants accurately stated that ADEs can be experienced by any age group and there was no statistically significant difference between HCPs and non-HCPs *(p* = 0.4). Overall, 80.9% (*n* = 157; *N* = 194) of non-HCPs and 92.8% (*n* = 90; *N* = 97) of HCPs were aware that anybody can report ADEs (*p* = 0.008).

**Fig. 3:**
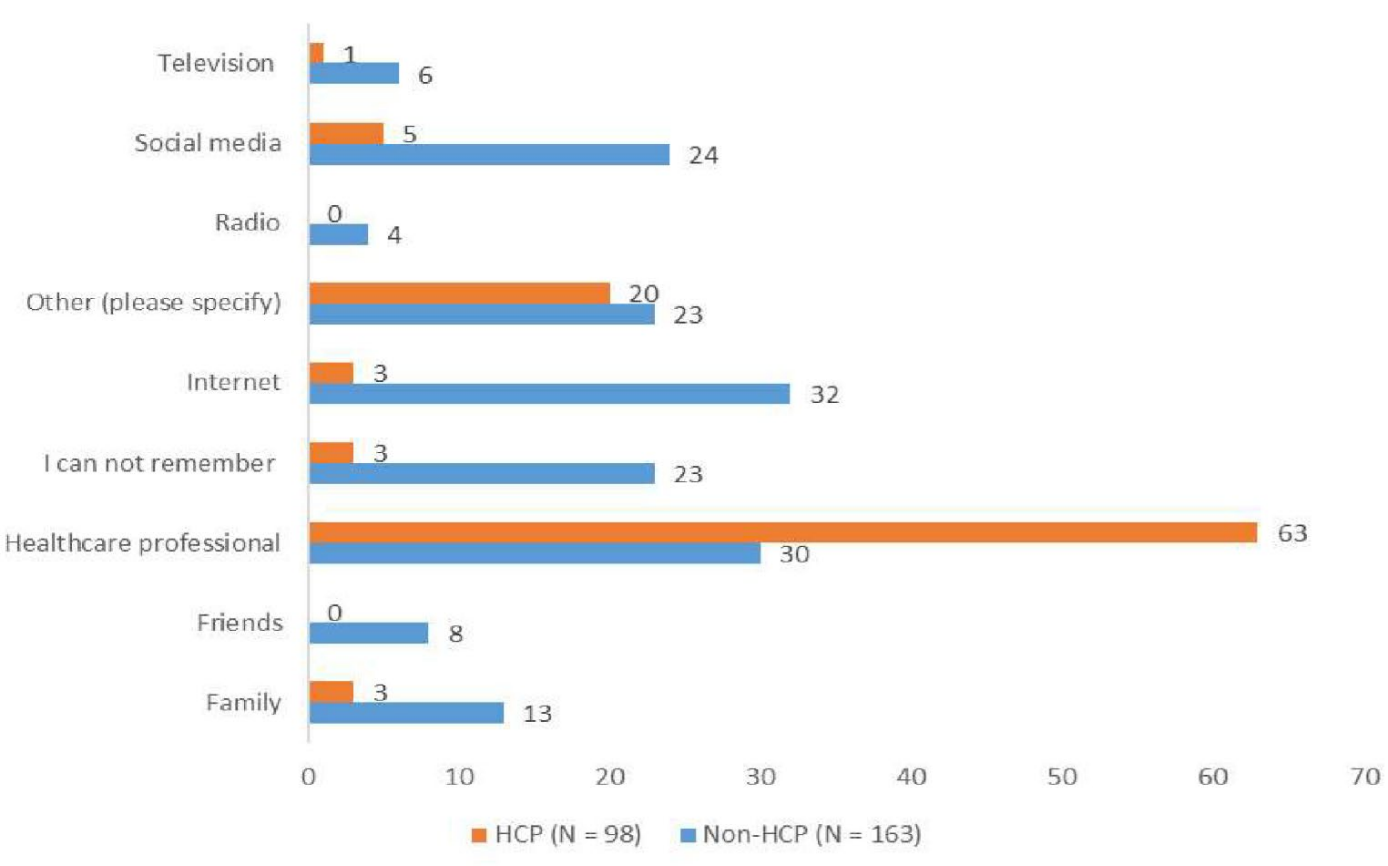
Participants’ sources of information for learning about ADE reporting. The category referred to as “other” includes work, school, and medicine package inserts

Regarding the requirement to report ADEs the majority of participants knew that ADEs needed to be reported, with 97.0% (*n* = 97; *N* = 100) HCPs and 82.7% (*n* = 167; *N* = 202) of non-HCPs knowing that ADEs had to be reported. This indicates that the HCPs were statistically more aware of ADEs and the need to report (*p* < 0.001). The majority knew that any person could report ADEs as indicated by 92.8% (*n* = 90; *N* = 97) of HCPs and 80.9% (*n* = 157; *N* = 194) of non-HCPs. Non-HCPs indicated that the internet was the source of information used to learn about reporting ADEs, whereas HCPs indicated that they learned from HCPs. Figure 3 depicts the various sources of information for learning about ADE reporting.

Overall, 77.4% (*n* = 260; *N* = 336) of the participants had heard of SAHPRA, with 92.9% (*n* = 92; *N* = 99) of the HCPs as opposed to 70.9% (*n* = 168; *N* = 237) of non-HCPs *(p* < 0.001). Awareness of the Med Safety App was lower among non-HCPs (8.4%, *n* = 20; *N* = 237) compared to HCPs (38.4%, *n* = 38; *N* = 99) *(p* < 0.001). Table 2 shows the obtained data on awareness criteria that were assessed during this study.

**Table 2:** Awareness of ADEs

Characteristic	*N*	Non-HCP^1^	HCP^1^	*p*-value
Has heard about the Med Safety app	336	*N* = 237 20 (8.4%)	*N* = 99 38 (38.4%)	< 0.001^*2*^
Has heard about SAHPRA	336	*N* = 237 168 (70.9%)	*N* = 99 92 (92.9%)	< 0.001^*2*^
Has heard about the term adverse drug events	350	*N* = 248 202 (81.5%)	*N* = 102 100 (98.0%)	< 0.001^*2*^
Know that adverse drug events should be reported	302	*N* = 202 167 (82.6%)	*N* = 100 97(97.0%)	< 0.001^*2*^
Know that all age groups can experience adverse drug events	291	*N* = 194 182 (93.8%)	*N* = 97 94 (96.9%)	0.4^*3*^
Aware that any person can report ADEs	291	*N* = 194 157 (80.9%)	*N* = 97 90 (92.8%)	0.008^*2*^

^*1*^ n (%)

^*2*^ Pearson’s Chi-squared test

^3^ Fisher’s exact test

*Note* HCP denotes participants who reported that they were healthcare professionals or who were enrolled in programs to become HCPs. Non- HCP refers to participants who are not healthcare professionals

### Attitudes Towards ADE and ADE Reporting

With slight variation (*p* = 0.7) between HCPs and non-HCPs, 97.9% (*n* = 329; *N* = 336) reported having used medicine in the past and the majority (90.1%, *n* = 290; *N* = 322) reported that they read the medication leaflet that came with their medicine. Some participants indicated that they did not the medication leaflet because it was not included with their medication.

Regarding the importance of reporting ADEs, 98% (*n* = 93; *N* = 95) of HCPs and 93% (*n* = 208; *N* = 224) of non-HCPs agreed that reporting was important (*p* = 0.075). The three key reasons why the participants felt (agree and strongly agree) it was important to report ADEs were indicated as making the HCP aware of what the medicine has caused (95.9% *n* = 306; *N* = 319); avoiding a repeat of the reaction in other people, (94.6%, *n* = 302; *N* = 319); and ensuring that the report reaches the medicine’s manufacturer (92.2%, *n* = 294; *N* = 319). Both the HCP and the non-HCP concur with these reasons. The least frequently cited reason why participants felt it was important to report ADEs was the desire to be compensated (25.7%, *n* = 82; *N* = 319). Table 3 presents the participant’s responses.

**Table 3:** Reason why reporting of ADEs is important

Participant response	Non-HCP *N* = 224^*1*^	HCP *N* = 95^*1*^
**To protect only oneself from harm**		
Strongly Disagree	39 (17.4%)	17 (17.9%)
Disagree	33 (14.7%)	13 (13.7%)
Uncertain	9 (4.0%)	0 (0%)
Agree	53 (23.7%)	18 (18.9%)
Strongly Agree	90 (40.2%)	47 (49.5%)
**To make the healthcare professional aware of what the medicine has caused**		
Strongly Disagree	6 (2.7%)	3 (3.2%)
Disagree	0 (0.0%)	0 (0.0%)
Uncertain	4 (1.8%)	0 (0.0%)
Agree	60 (26.8%)	17 (17.9%)
Strongly Agree	154 (68.8%)	75 (78.9%)
**To make sure the medicine is not fake**		
Strongly Disagree	8 (3.6%)	6 (6.3%)
Disagree	14 (6.3%)	9 (9.5%)
Uncertain	30 (13.4%)	15 (15.8%)
Agree	60 (26.8%)	19 (20.0%)
Strongly Agree	112 (50.0%)	46 (48.4%)
**To ensure that the healthcare professional is reprimanded.**		
Strongly Disagree	24 (10.7%)	34 (35.8%)
Disagree	47 (21.0%)	26 (27.4%)
Uncertain	49 (21.9%)	13 (13.7%)
Agree	53 (23.7%)	9 (9.5%)
Strongly Agree	51 (22.8%)	13 (13.7%)
**To avoid a repeat of the reaction in other people**		
Strongly Disagree	6 (2.7%)	1 (1.1%)
Disagree	1 (0.4%)	1 (1.1%)
Uncertain	6 (2.7%)	2 (2.1%)
Agree	64 (28.6%)	18 (18.9%)
Strongly Agree	147 (65.6%)	73 (76.8%)
**To ensure that the report reaches the medicine’s manufacturer**		
Strongly Disagree	6 (2.7%)	0 (0.0%)
Disagree	2 (0.9%)	0 (0.0%)
Uncertain	11 (4.9%)	6 (6.3%)
Agree	52 (23.2%)	14 (14.7%)
Strongly Agree	153 (68.3%)	75 (78.9%)
**To make sure the medicine’s manufacturer stops making the medicine**		
Strongly Disagree	15 (6.7%)	10 (10.5%)
Disagree	50 (22.3%)	28 (29.5%)
Uncertain	60 (26.8%)	27 (28.4%)
Agree	36 (16.1%)	12 (12.6%)
Strongly Agree	63 (28.1%)	18 (18.9%)
**To ensure that you get compensated for the experienced adverse drug event**		
Strongly Disagree	35 (15.6%)	29 (30.5%)
Disagree	56 (25.0%)	28 (29.5%)
Uncertain	67 (29.9%)	22 (23.2%)
Agree	33 (14.7%)	11 (11.6%)
Strongly Agree	33 (14.7%)	5 (5.3%)

^*1*^ n (%)

*Note* HCP denotes participants who reported that they were healthcare professionals or who were enrolled in programs to become HCPs. Non- HCP refers to participants who are not healthcare professionals

When asked if they had asked their healthcare provider regarding ADEs, 52.4% (*n* = 118; *N* = 225) of non-HCPs and 45.9% (*n* = 45; *N* = 98) of HCPs responded that they did (*p* = 0.3). Altogether 51.1% (*n* = 115; *N* = 225) of non-HCPs were provided information about the potential ADEs by their HCPs, as opposed to 66.3% (*n* = 65; *N* = 98) of HCPs (*p* = 0.011). Not receiving medicines from HCPs was one aspect that contributed to not obtaining information from the HCP, with nearly 18.7% (*n* = 42; *N* = 225) of non-HCPs and 10.2% (*n* = 10; *N* = 98) of HCPs obtaining their medicine from other sources (*p* = 0.057). Table 4 shows the obtained data on medicine user attitudes.

**Table 4:** Attitudes on ADE and ADE reporting

Characteristic	*N*	Non-HCP^1^	HCP^1^	*p*-value
History of medication use	336	*N* = 237 231 (97.5%)	*N* = 99 98 (99.0%)	0.7^*3*^
Asked the health professional about ADEs	323	*N* = 225 118 (52.4%)	*N* = 98 45 (45.9%)	0.3^2^
Read the medication leaflet	322	*N* = 225 200 (88.9%)	*N* = 97 90 (92.8%)	0.3^*2*^
Healthcare professionals provided information on ADEs	323	*N* = 225 115 (51.1%)	*N* = 98 65 (66.3%)	0.011^*2*^
Medicine not obtained from health professional	323	*N* = 225 42 (18.7%)	*N* = 98 10.2 (10%)	0.057^*2*^
Believe it is important or necessary to report ADEs	319	*N* = 224 208 (92.9%)	*N* = 95 93 (97.9%)	0.075^*2*^

^*1*^ n (%)

^*2*^ Pearson’s Chi-squared test

^3^ Fisher’s exact test

*Note* HCP denotes participants who reported that they were healthcare professionals or who were enrolled in programs to become HCPs. Non- HCP refers to participants who are not healthcare professionals

**Fig. 4:**
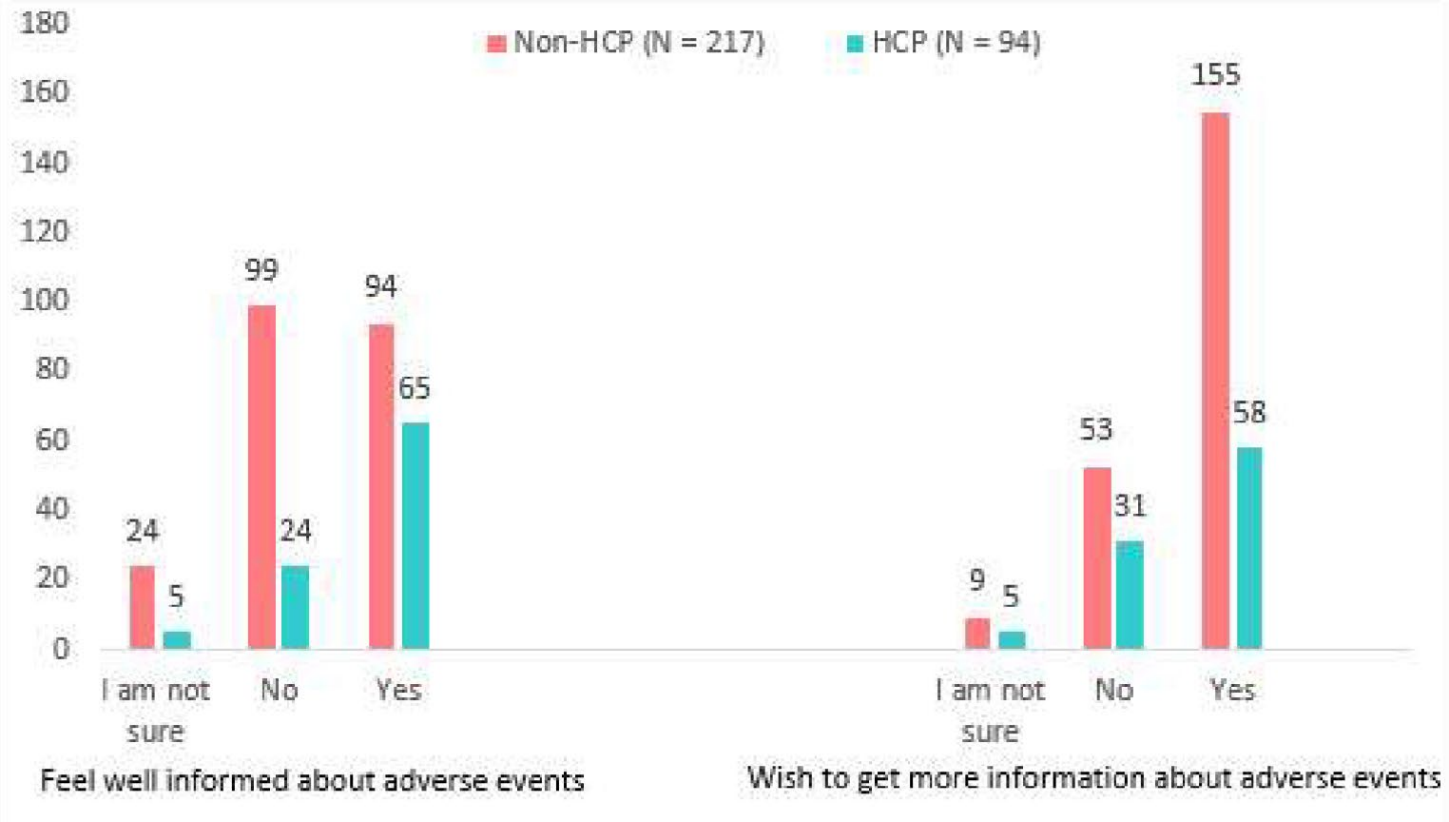
Participants’ current knowledge on, and desire to learn about ADEs

Overall, 45.6% (*n* = 99; *N* = 217) of non-HCPs did not feel well informed about the adverse effects that their medication may cause, compared with 25.5% (*n* = 24; *N* = 94) of the HCPs. A larger percentage (71.4%, *n* = 155; *N* = 217) of non-HCPs expressed a wish to learn more about the side effects of their medicine compared with 61.7% (*n* = 58; *N* = 94) of the HCPs. The responses that were received are presented in Fig. 4.

## Discussion

Study participants were aware of ADEs with 86.3% having heard about the term ADEs. Most participants (94.8%) knew that ADEs can be experienced by any age group, 87.4% of participants knew that ADEs had to be reported and 84.9% were aware that anybody can report ADEs. This awareness among participants can be explained by the fact that 85.8% of participants had attained tertiary education and 91.5% resided in urban areas. It has been shown that participants with higher education and those who live in urban areas have higher levels of awareness of ADEs [6]. With 90.1% of participants having read the medication leaflet, this heightened awareness is to be expected. Similar results were obtained from a study done in South Africa which reported that 70.9% of participants were aware of ADEs [12]. According to comparable studies, between 25.7% and 51.9% of participants could correctly define an ADE [19–22], with up to 32.2% of participants not knowing about ADEs, according to one study [27]. Awareness regarding ADEs was significantly higher (*p* < 0.001) in the HCPs and this is aligned with previous studies, which also found awareness regarding ADEs and pharmacovigilance to be high amongst HCPs [28–30].

Only 8.4% of the non-HCPs knew about the Med Safety App. The Med Safety App was launched in South Africa in 2021 and enables users to report suspected ADEs [31]. Despite marketing efforts made to increase awareness of the Med Safety App at its launch [32, 33] it is important to sustain these efforts to improve awareness and enhance the reporting of ADEs in South Africa. Awareness of Med Safety App was significantly higher among the HCPs (*p* < 0.001), however only 38.4% knew about the existence of this tool. This lack of awareness regarding the reporting process or tools has also been noted as a barrier to reporting of ADEs by HCPs in other studies [13, 34, 35]. This indicates that efforts aimed at consumers to improve reporting of ADEs must also be extended to HCPs [20]. Similar to our findings, studies conducted in Ghana and Nigeria on consumers’ awareness of ADE reporting tools also observed insufficient awareness [6, 18]. This highlights the importance of raising consumers’ awareness of reporting tools after they are developed.

As expected, knowledge of SAHPRA by participants was lower among non-HCPs compared to HCPs (70.9% vs. 92.9%; *p* < 0.001) because HCPs can interact with SAHPRA on other topics other than ADE reporting. In South Africa all patient information leaflets must contain a statement that informs readers that they can report any side effects to SAHPRA if they occur [36]. With 90.1% of the participants having read the medication leaflet, this heightened awareness is to be anticipated.

The main source of information on reporting of ADEs for the HCPs was other HCPs, while for non-HCPs the main source was the internet. This is troubling because 81.8% of non-HCPs who reported a history of medicine use acquired the medicines from a HCP, indicating that consumers may not be actively informed about ADEs during interactions with HCPs. This finding is similar to another study in which only 29.5% of HCPs encouraged patients to report ADEs [21].

The majority of HCPs (98%) and non-HCPs (93%) agreed it was important to report ADEs. This finding is encouraging because it increases the likelihood that campaigns to promote the reporting of ADEs may be well received. Similar conclusions regarding the significance of reporting ADEs were found in comparable research involving consumers [12, 18, 19, 21]. However, it must be noted that high awareness and positive attitudes regarding the reporting of ADEs does not always influence reporting practices [12, 37]. This discrepancy is evident in the global challenge of ADE underreporting [14–16].

The most common reasons given by both HCPs and non-HCPs for thinking it is essential to report ADEs were to ensure that the HCP is made aware of what the medicine has caused, to avoid the same reaction in other people, and to ensure that the report reaches the medicine’s manufacturer. This communalist mind-set was also seen in similar studies [12, 19, 38–42]. However, 68.4% of HCP and 63.8% of non-HCP indicated that reporting could be done only to protect oneself from harm.

Fear of litigation is frequently cited as a deterrent to ADE reporting by HCPs [6, 19, 34, 35, 37] and this fear may not be unfounded as for 46.4% of non-HCPs and 23.2% of HCPs ADE reporting was important to ensure that the healthcare provider is reprimanded. For 76.8% of non-HCPs and 68.4% of HCPs, ADE reporting was important to assure themselves of the medicine’s authenticity. This concern is justified because fake medications pose a serious risk to public safety in developing nations and lead to the use of ineffective medications and subsequent treatment failures [43].

Our results show that 45.6% of non-HCPs and 25.5% of HCPs did not feel well informed about the adverse effects that their medication may cause. According to a scoping study conducted across six countries, patients requested information on ADEs in addition to information on medicine use and storage [44]. Having a setting where patients feel knowledgeable and assured is an important factor that may lead to increased public involvement in pharmacovigilance [45]. Therefore, it is essential to improve interactions between patients and HCPs so that information about ADEs can be shared. Because it is assumed that HCPs have access to more resources to obtain additional information, they may need regarding their medication, it is concerning that 25.5% of HCPs felt under-informed about the potential side effects of their medication. It is encouraging that most participants expressed a desire to learn more about the side effects of their medication, demonstrating a willingness to learn and adapt practices in relation to ADEs.

Almost all the participants, 97.9%, reported having used medicines. Receiving guidance from HCPs regarding potential ADEs and the need to report them is one of the cited reasons that ADEs are reported [38, 46]. Only 51.5% of non-HCPs stated that the healthcare provider had provided them with information on ADEs. Strategies to improve communication regarding ADEs during health seeking interactions may favorably influence ADE reporting given that the internet and social media are largely responsible for the non-HCPs’ knowledge that ADEs are reportable. Patient-initiated communication should also be emphasised in these communication strategies as only 52.4% of the participants in this study reported to have asked their HCP about ADEs, this is lower that the results obtain in another study which reported 64% [21]. Regarding the history of medication use, reading the medication leaflet, and belief that reporting ADEs is important or necessary, there was no discernible difference between the HCP and non-HCPs. Unexpectedly 66.3% of the HCPs indicated that they obtained information on ADEs from their healthcare providers compared to 51.5% of the non-HCP. This finding suggests that healthcare providers need to be encouraged to provide information on ADEs more often to their patients.

A strong pharmacovigilance program must be supported by a drug safety awareness culture where all stakeholders including healthcare providers and users of medicines are confident in their understanding of the benefits and risks associated with the products they recommend, prescribe, or use [47].

The fact that only participants who viewed the Netwerk24/News24 platform or had access to the News24 platform during the time of the advertisement were invited to participate in the study is a limitation of the recruitment and data collection strategy employed in this study. Another limitation of the study was that the data collection instrument was only available on an online platform which required internet access. This could have led to more participants being from the urban areas. As the data collection instrument was only available in English only participants with sufficient proficiency in English could complete the questionnaire. In addition, the relatively small sample size and the predominance of respondents from urban areas represents a limitation although participants were drawn from all nine provinces of South Africa. It is likely that participants may have been more aware of ADEs which limits the generalisability of the findings of this study. Finally, the study may have been subject to recall bias, as it relied on participants’ ability to accurately remember and report past experiences. This could have influenced the reliability of the data collected.

Despite the limitations outlined above, the study successfully met its objectives and contributed valuable insights into the current state of adverse drug event (ADE) reporting in South Africa. The perspectives of healthcare professionals, in their role as medicine users, provided meaningful information that can inform future initiatives aimed at improving patient involvement in ADE reporting within the country.

## Conclusion

Adult South Africans are aware of ADEs and ADE reporting. There is a positive attitude towards reporting of ADEs, with participants agreeing on the importance of reporting ADEs. The optimistic outlook seen in this study should be leveraged to support initiatives that encourage the reporting of ADEs in South Africa. Both HCPs and non-HCPs should be the target of these activities.

## Data Availability

The data that support the findings of this study are available on request from the corresponding author. The data are not publicly available due to privacy or ethical restrictions.

## References

[1] WHO (World Health Organization). The safety of medicines in public health programmes: pharmacovigilance an essential tool. WHO Press. 2006. Accessed 20 Aug. 2023. https://apps.who.int/iris/handle/10665/43384

[2] Hazell L, Cornelius V, Hannaford P, Shakir S, Avery AJ, Yellow Card Study C. How do patients contribute to signal detection? A retrospective analysis of spontaneous reporting of adverse drug reactions in the UK’s yellow card scheme. Drug Saf. 2013;36(3):199–206. 10.1007/s40264-013-0021-2. 23444232 10.1007/s40264-013-0021-2

[3] ENCePP (European Network of Centres for Pharmacoepidemiology and Pharmacovigilance). Guide on Methodological Standards in Pharmacoepidemiology. Accessed 17 Aug 2023. https://www.encepp.eu/standards_and_guidances/methodologicalGuide7_4.shtml

[4] Inacio P, Cavaco A, Airaksinen M. The value of patient reporting to the pharmacovigilance system: a systematic review. Br J Clin Pharmacol. 2017;83(2):227–46. 10.1111/bcp.13098. 27558545 10.1111/bcp.13098 PMC5237689

[5] Harmark L, van Hunsel F, Grundmark B. ADR reporting by the general public: lessons learnt from the Dutch and Swedish systems. Drug Saf. 2015;38(4):337–47. 10.1007/s40264-015-0264-1. 25627832 10.1007/s40264-015-0264-1

[6] Jacobs TG, Hilda Ampadu H, Hoekman J, Dodoo ANO, Mantel-Teeuwisse AK. The contribution of Ghanaian patients to the reporting of adverse drug reactions: a quantitative and qualitative study. BMC Public Health. 2018;18(1):1384. 10.1186/s12889-018-6285-9. 30563498 10.1186/s12889-018-6285-9 PMC6299566

[7] Matlala MF, Lubbe MS, Steyn H. The completeness of adverse drug reaction reports in South Africa: an analysis in VigiBase(R). Afr J Prim Health Care Fam Med. 2023;15(1):1–a93659. 10.4102/phcfm.v15i1.3659. 10.4102/phcfm.v15i1.3659 PMC9900287 36744452

[8] SAHPRA (South African Health Products Regulatory Authority). VigiGuardian newsletter. Accessed 22. Jun. 2023, https://www.sahpra.org.za/document/vigiguardian-newsletter-april-2022/

[9] WHO (World Health Organization). The WHO Programme for International Drug Monitoring. Accessed 17. Aug. 2023, https://www.who.int/teams/regulation-prequalification/regulation-and-safety/pharmacovigilance/networks/pidm

[10] Medicines. and Related Substances Act, (1965).

[11] SAHPRA (South African Health Products Regulatory Authority). Guideline for adverse drug reactions (ADRs) reporting for healthcare professionals. 2022. Accessed 9 Sep. 2023. https://www.sahpra.org.za/wp-content/uploads/2022/08/SAHPGL-CEM-PV-06-v2_Adverse-Drug-Reactions-ADRs-Reporting-for-Healthcare-Professionals.pdf

[12] Pillay S, Mulubwa M, Viljoen M. Parental reporting of adverse drug reactions in South Africa: an online survey. Afr J Prim Health Care Fam Med. 2021;13(1):e1–8. 10.4102/phcfm.v13i1.2880. 34636609 10.4102/phcfm.v13i1.2880 PMC8517735

[13] Varallo FR, Guimaraes Sde O, Abjaude SA, Mastroianni Pde C. Causes for the underreporting of adverse drug events by health professionals: a systematic review. Rev Esc Enferm USP. 2014;48(4):739–47. 10.1590/s0080-623420140000400023. Causes del subregisto de los eventos adversos de medicamentos por los profesionales de la salud: revision sistematica. 25338257 10.1590/s0080-623420140000400023

[14] Alatawi YM, Hansen RA. Empirical Estimation of under-reporting in the U.S. Food and drug administration adverse event reporting system (FAERS). Expert Opin Drug Saf Jul. 2017;16(7):761–7. 10.1080/14740338.2017.1323867. 10.1080/14740338.2017.1323867 28447485

[15] Deslandes PN, Bracchi R, Jones K, et al. Changes in suspected adverse drug reaction reporting via the yellow card scheme in Wales following the introduction of a National reporting Indicator. Br J Clin Pharmacol Aug. 2022;88(8):3829–36. 10.1111/bcp.15326. 10.1111/bcp.15326 PMC9544666 35322450

[16] Fernandez S, Lenoir C, Samer C, Rollason V. Drug interactions with Apixaban: A systematic review of the literature and an analysis of vigibase, the world health organization database of spontaneous safety reports. Pharmacol Res Perspect Oct. 2020;8(5):e00647. 10.1002/prp2.647. 10.1002/prp2.647 PMC7507549 32881416

[17] SAHPRA (South African Health Products Regulatory Authority). SAHPRA Launches The Med Safety App For Self-Reporting Of Suspected Adverse Drug Reactions By The Public And Healthcare Professionals. Accessed 09. Sep 2023. https://www.sahpra.org.za/press-releases/sahpra-launches-the-med-safety-app-for-self-reporting-of-suspected-adverse-drug-reactions-by-the-public-and-healthcare-professionals/

[18] Adisa R, Adeniyi OR, Fakeye TO. Knowledge, awareness, perception and reporting of experienced adverse drug reactions among outpatients in Nigeria. Int J Clin Pharm. 2019;41(4):1062–73. 10.1007/s11096-019-00849-9. 31140162 10.1007/s11096-019-00849-9

[19] Kim S, Yu YM, You M, Jeong KH, Lee E. A cross-sectional survey of knowledge, attitude, and willingness to engage in spontaneous reporting of adverse drug reactions by Korean consumers. BMC Public Health. 2020;20(1):1527. 10.1186/s12889-020-09635-z. 33032559 10.1186/s12889-020-09635-z PMC7545860

[20] Sabblah GT, Darko DM, Mogtari H, Harmark L, van Puijenbroek E. Patients’ perspectives on adverse drug reaction reporting in a developing country: a case study from Ghana. Drug Saf. 2017;40(10):911–21. 10.1007/s40264-017-0563-9. 28653291 10.1007/s40264-017-0563-9

[21] Sales I, Aljadhey H, Albogami Y, Mahmoud MA. Public awareness and perception toward adverse drug reactions reporting in Riyadh, Saudi Arabia. Saudi Pharm J. 2017;25(6):868–72. 10.1016/j.jsps.2017.01.004. 28951672 10.1016/j.jsps.2017.01.004 PMC5605842

[22] Wang N, Chen Y, Ren B, et al. A cross-sectional study: comparison of public perceptions of adverse drug reaction reporting and monitoring in Eastern and Western China. BMC Health Serv Res. 2022;22:318. 10.1186/s12913-022-07720-0. 35260158 10.1186/s12913-022-07720-0 PMC8905784

[23] News24. Give your opinion about medication. Accessed 01. Oct 2023, 2023. https://www.news24.com/news24/partnercontent/give-your-opinion-about-medication-20230306

[24] SurveyMonkey Inc. SurveyMonkey. Accessed 09 Sep 2023. 2023. https://www.surveymonkey.com/

[25] Interactive Advertising Bureau. Top Rankings. Accessed 27. Jan 2025, https://lookerstudio.google.com/u/0/reporting/d20f287c-b84c-4278-a6a7-64fccf8badbc/page/p_u1t1n7pfld

[26] R Foundation. The R project for statistical computing. Accessed 11. Nov 2023, https://www.r-project.org/

[27] Patel JJ, Shah MK, Patel PP, Gandhi AM, Desai MK. Knowledge, attitude and practice among consumers about adverse drug reaction reporting. Int J Basic Clin Pharmacol. 2019;8(8):1776–82. 10.18203/2319-2003.ijbcp20193177.

[28] Gordhan A, Bangalee V. The current knowledge, attitude, perceptions and practice of pharmacovigilance amongst community pharmacists in Gauteng, South Africa. J Pharm Health Serv Res. 2022;13(2):73–82. 10.1093/jphsr/rmac012.

[29] Hussain R, Hassali MA, Hashmi F, Akram T. Exploring healthcare professionals’ knowledge, attitude, and practices towards pharmacovigilance: a cross-sectional survey. J Pharm Policy Pract. 2021;14(1):5. 10.1186/s40545-020-00287-3. 33397478 10.1186/s40545-020-00287-3 PMC7784002

[30] Khan Z, Karatas Y, Hamid SM. Evaluation of health care professionals’ knowledge, attitudes, practices and barriers to pharmacovigilance and adverse drug reaction reporting: a cross-sectional multicentral study. PLoS One. 2023;18(5):e0285811. doi:10.1371/journal.pone.0285811. 10.1371/journal.pone.0285811 PMC10208525 37224133

[31] SAHPRA (South African Health Products Regulatory Authority). The Med Safety App. Accessed 09 Sep 2023. 2023. https://medsafety.sahpra.org.za/

[32] DOH (Department of Health). Sahpra Med Safety Mobile App. Accessed 09 Sep 2023. 2023. https://sacoronavirus.co.za/2021/09/02/sahpra-med-safety-mobile-app/

[33] News24. App allows users to report adverse effects from medicines, including Covid-19 vaccines. Accessed 09. Sep 2023, 2023. https://www.news24.com/life/wellness/body/condition-centres/infectious-diseases/coronavirus/app-allows-users-to-report-adverse-effects-from-medicines-including-covid-19-vaccines-20210422

[34] Bogolubova S, Padayachee N, Schellack N. Knowledge, attitudes and practices of nurses and pharmacists towards adverse drug reaction reporting in the South African private hospital sector. Health SA Gesondheid. 2018;23:10641064. 10.4102/hsag.v23i0.1064. 10.4102/hsag.v23i0.1064 PMC6917443 31934371

[35] Le TT, Nguyen TTH, Nguyen C, et al. Factors associated with spontaneous adverse drug reaction reporting among healthcare professionals in Vietnam. J Clin Pharm Ther. 2020;45(1):122–7. 10.1111/jcpt.13037. 31486525 10.1111/jcpt.13037

[36] SAHPRA (South African Health Products Regulatory Authority). Guideline for patient information leaflet for human-medicines categories A-D. Vol. 2023. 2022. Accessed 9 Sep. 2023. https://www.sahpra.org.za/document/guideline-for-patient-information-leaflet-for-human-medicines-categories-a-d/

[37] Asiamah M, Akuffo KO, Nortey P, Donkor N, Danso-Appiah A. Spontaneous reporting of adverse drug reaction among health professionals in Ghana. Arch Public Health. 2022;80(1):3333. 10.1186/s13690-021-00783-1. 10.1186/s13690-021-00783-1 PMC8772084 35057859

[38] Al Dweik R, Yaya S, Stacey D, Kohen D. Factors affecting patient reporting of adverse drug reactions: a systematic review. Br J Clin Pharmacol. 2017;83875–83. 10.1111/bcp.13159. 10.1111/bcp.13159 PMC5346870 27868226

[39] Hariraj V, Aziz Z. Patient reporting of adverse drug reactions (ADRs): survey of public awareness and predictors of confidence to report. Ther Innov Regul Sci. 2018;52(6):757–63. 10.1177/2168479017745025. 29714567 10.1177/2168479017745025

[40] Kitisopee T, Assanee J, Sorofman BA, Watcharadmrongkun S. Consumers’ adverse drug event reporting via community pharmacists: three stakeholder perception. J Pharm Policy Pract. 2022;15:1–10. 10.1186/s40545-022-00417-z. 35287746 10.1186/s40545-022-00417-z PMC8919562

[41] Valinciute-Jankauskiene A, Loreta K. Qualitative study of general public views towards adverse drug reactions in Lithuania. Healthc (Basel). 2021;9(3):303. 10.3390/healthcare9030303. 10.3390/healthcare9030303 PMC8001150 33803215

[42] van Hunsel F, Harmark L, Pal S, Olsson S, van Grootheest K. Experiences with adverse drug reaction reporting by patients an 11-country survey. Drug Saf. 2012;35(1):45–60. 10.2165/11594320-000000000-00000. 22149419 10.2165/11594320-000000000-00000

[43] SAHPRA (South African Health Products Regulatory Authority). Fake medicines are a dangerous threat in Africa: 3 ways to spot them. Accessed 17. Sep 2023, 2023. https://www.sahpra.org.za/news-and-updates/fake-medicines-are-a-dangerous-threat-in-africa-3-ways-to-spot-them/

[44] Kusch MK, Haefeli WE, Seidling HM. How to Meet patients’ individual needs for drug information - a scoping review. Patient Prefer Adherence. 2018;12:2339–55. 10.2147/PPA.S173651. 30464421 10.2147/PPA.S173651 PMC6229142

[45] Schurer MJ, Bam L, De Kock I. An investigation into the value of a standardised global pharmacovigilance reporting system. S Afr J Ind Eng. 2017;28(3):78–88. 10.7166/28-3-1841.

[46] Zondi S, Naidoo P. Perceptions, practices and barriers to reporting of adverse drug reactions among HIV infected patients and their Doctors in 3 public sector hospitals of the ethekwini metropolitan, Kwa-Zulu Natal: a cross sectional and retrospective analysis. BMC Health Serv Res. 2022;22(1):1054. 10.1186/s12913-022-08395-3. 35982442 10.1186/s12913-022-08395-3 PMC9389709

[47] Mehta U, Kalk E, Boulle A, et al. Pharmacovigilance: a public health priority for South Africa. S Afr Health Rev. 2017;2017:125–33. 29200789 PMC5708547

